# Management of necrotizing fasciitis and the use of sealed irrigation system: A case report

**DOI:** 10.1016/j.amsu.2022.103432

**Published:** 2022-03-03

**Authors:** Ayham Sammoni, Okbah Mohamad, Ali Abdalah, Monaf Borhan Alghazali, Maen Al-Aissami

**Affiliations:** Department of Plastic and Reconstructive Surgery, Al Mouwasat University Hospital, Faculty of Medicine, Damascus University, Damascus, Syria

**Keywords:** Necrotizing fasciitis, Sealed irrigation system, Debridement, Case report

## Abstract

**Introduction:**

Necrotizing fasciitis [NF] is a deep soft tissue infection with high morbidity and mortality. The diagnosis is mainly clinical, and is confirmed during the surgical exploration, which is also the cornerstone of treatment.

**Case presentation:**

We report a case of a 16-year-old female who complained of pain, tenderness, and erythema along her abdomen and back after a minor trauma on her left palm. The patient was treated with systemic antibiotics and daily surgical debridement followed by placement of a bilateral suction drainage system. A split-thickness graft was used to cover the defect on the lower back of the patient.

**Conclusion:**

Sealed irrigation technique is an effective method to manage NF. Furthermore, it reduces the hospitalization duration by continuously removing the necrotic and infected material that hinder tissue healing.

## Highlights

- Necrotizing fasciitis is a rapidly spreading and fatal disease
- Early surgical exploration and debridement are the cornerstones of the treatment of necrotizing fasciitis.
- The sealed irrigation technique is effective in treating necrotizing fasciitis.

## 1 Introduction

Necrotizing fasciitis (NF) is a deep soft tissue infection that causes progressive destruction of the muscle fascia and overlying subcutaneous fat [1]. Because of the high morbidity and mortality [2], early diagnosis and treatment with adequate antibiotics and surgical intervention are vital [3]. Although NF can occur in the absence of clinical risk factors [4], diabetes mellitus, age >60 years, obesity, underlying malignancy, and chronic disorders are documented risk factors [5]. Multiple approaches to diagnose and treat NF were described, with early surgical exploration and debridement being the cornerstone of treatment [6]. We present a case of NF of the back and abdomen in a young healthy girl who was effectively treated with a new approach tailored to our hospital's needs. This case report has been reported in line with the SCARE Criteria [7].

## 2 Case presentation

A 16-year old woman presented to the emergency department (ED) at Al-Mouwasat University hospital on July 12, 2021 with pain, tenderness, and erythema along her back and abdomen after a minor trauma on her left palm on July 9, 2021.

Initially, she suffered from enlarged masses on her cubital fossa followed by pain and tenderness with erythema along her left axilla. The same symptoms developed on both flanks, lower abdomen, and back in the following three days while subsided from the arm.

She had no previous medical illness. On the physical examination, the patient looked tired. Her vital signs were: Body temperature: 36, pulse: 121, respiratory rate: 36, SO2: 98%, blood pressure: 10/7. On inspection: Her skin appeared normal to mild erythematous. No crepitations were palpated. Ultrasonography showed three reactive lesions on the axilla with no lymphadenopathy.

**Fig. 1:**
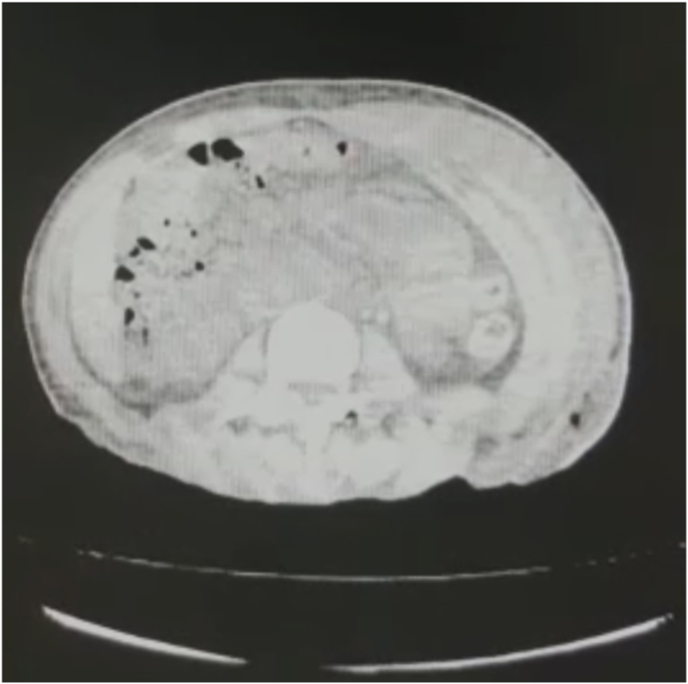
Computed tomography showing gas and edema in subcutaneous and deep fascial plains of back and abdomen.

The patient was admitted to the infectious disease department and was treated with empirical antibiotics [clindamycin, vancomycin, ceftazidime, fluconazole] after taking a blood sample for culture for suspected acute cellulitis for 3 days without improvement. Computed tomography (CT) without contrast of the chest and abdomen showed gas and edema extending along the deep fascial planes and superficial muscles of the back and abdomen (Fig. 1). After plastic surgery consultation, the patient was prepared to undergo emergent surgery for the management of presumed necrotizing fasciitis.

**Fig. 2:**
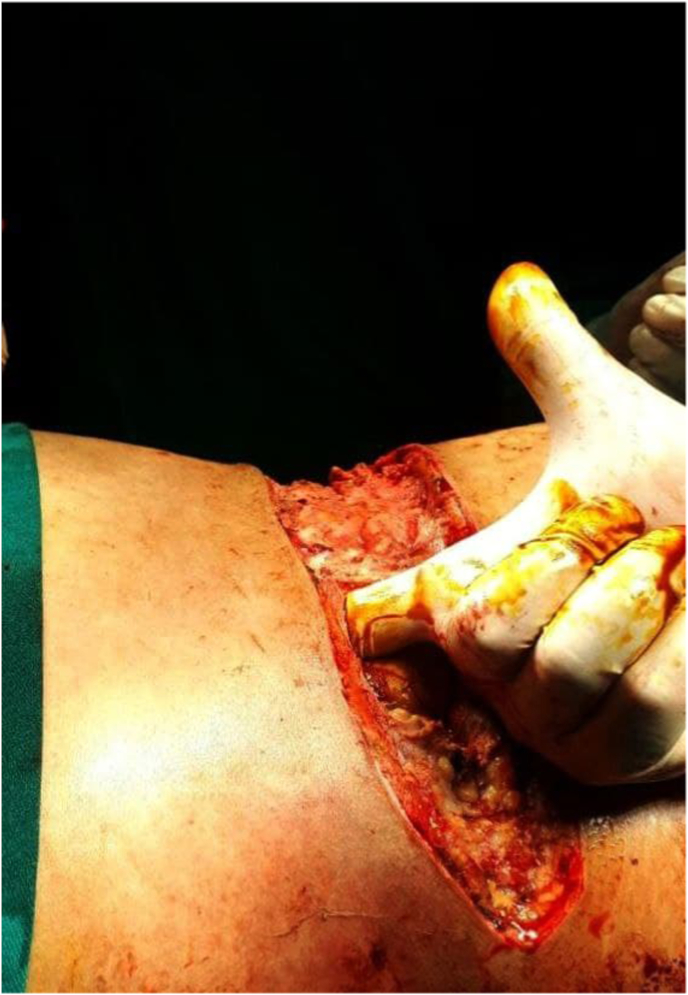
Debridement of the skin of the lower back and blunt dissection.

In the operation room (OR), the plastic surgeons found a foul-smelling purulent discharge and extensive tissue necrosis after appropriate incisions on the lower back and lower abdomen. Blunt dissection is performed easily using the index finger between the subcutaneous tissue and the deep fascia and a wide space was detected extending superiorly from the lower back to the posterolateral thoracic wall and axilla and also from the lower abdominal wall to the umbilical region. The necrotized skin at the lower back was debrided (Fig. 2). Pathology of the skin and subcutaneous tissue was consistent with acute inflammation and necrosis.

All the cavities were copiously irrigated with normal saline, debrided, and packed with sterilized gauze after taking a tissue biopsy for pathological and culture and sensitivity studies.

Blood culture on admission revealed multidrug sensitive group A Streptococcus (GAS). Therefore, the internists changed the antibiotic regimen to guide against GAS [Tazopactam/Piperilline, Tigecycline].

The patient showed improvement after the first surgery and was scheduled to perform daily surgical exploration for more debridement and gauze replacement for a week.

On July 24, 2021, we closed the lower abdominal wound and used a bilateral suction drainage system connected to the closed deep fascial plain. One to the right abdomen and the other to the left flank and abdomen. This system consisted of an irrigating tube that was flushed with normal saline every 3 hours and then the flushing liquid was drained through suction drainage in a way we called sealed irrigation technique. We did this procedure for ten days to ensure a clear irrigation solution.

**Fig. 3:**
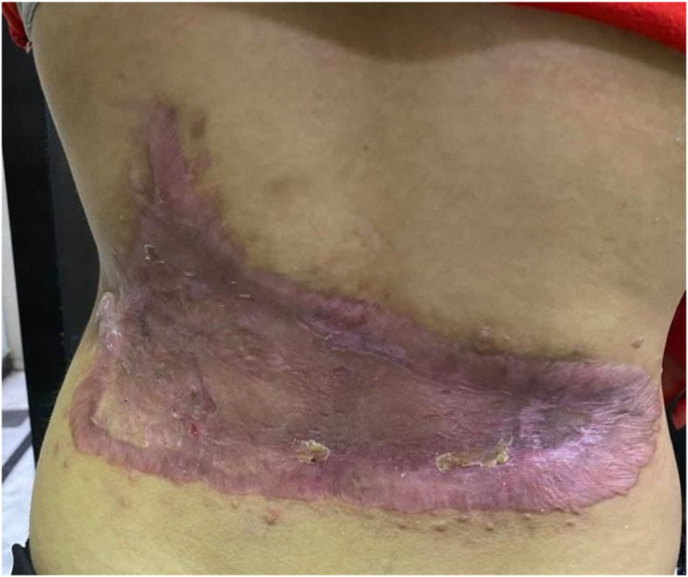
3- Months post reconstruction with skin grafts.

Afterwards, a healthy clean granulation tissue developed along the discovered spaces, and a split-thickness graft was taken from the lateral side of the thigh and meshed to cover opened wound at the lower back. Unfortunately, the graft was lost due to nosocomial pseudomonas infection. We applied acetic acid 3% and polymyxin ointment for a week on the recipient site on the lower back, and a new skin biopsy was performed for culture which was negative. A second split-thickness graft from the lateral side of the same thigh was used to reconstruct the lower back which was successful (Fig. 3). The patient and his parents were satisfied with the results and discharged from the hospital and maintained scheduled follow-up visits.

## 3 Discussion

NF has mainly been recognized with two types: Type 1—accounts for the majority of cases and has a polymicrobial origin, and Type 2—has a monomicrobial origin [group A Streptococcus, methicillin-resistant *Staphylococcus aureus*] [6]. Unlike type I infections, Type II infections can develop at any age group, in people without any medical history, and without an overt lesion [1,6]. In this case, the 16-year old girl suffered from GAS NF of her back and abdomen following minor trauma to her palm, which later spread to her back and abdomen most likely via a hematogenous route [6,8,9].

The course of Type 2 is aggressive, with accelerated systemic decline [10]. Severe signs and symptoms may not appear until considerable tissue damage has occurred and the patient is already at high risk for an unfavorable outcome [1].

The most frequent sign reported at the time of presentation is pain out of proportion [8], which is a deciding factor in distinguishing NF from cellulitis combined with recent surgery, hypotension, skin necrosis, fluctuance, diarrhea, and hemorrhagic bullae [11]. The former factors were missing in this case, which further complicated and prolonged the surgical diagnosis and treatment.

Imaging studies [Plain X-rays, computerized tomography, and magnetic resonance imaging] are useful in determining the presence and extent of infection [8], though they fall short of establishing the definitive diagnosis, which is largely dependent on surgical exploration.

A culture and Gram stain of specimens obtained from the deep tissue during surgical exploration, or positive blood cultures, are used to identify the responsible pathogen [1].

Prompt surgical exploration and debridement of the necrotic tissue are the gold standard treatments when NF is either suspected or diagnosed [1]. Initial tissue findings may include gray necrotic tissue, lack of bleeding, “dishwater” pus, and a positive “finger test” result, which is characterized by lack of resistance to finger dissection in typically adherent tissues [12].

The patient should undergo a relook surgery within 24 hours of the initial intervention for subsequent debridement [13]. This should be done every day until the surgical team is satisfied that all necrotic tissue has been eradicated and only healthy tissue remains [1].

The used method in this study is not novel. It was published in January 2016 and updated by En-Hui Zhou et al., in 2020 as double-cannula irrigation–drainage tube [14]. The main advantage of this technique is to obtain the necrotic material through the suction tube. However, due to the lack of this equipment in our country, we established this technique using two single tubes, one to irrigate and the other to drain.

We relied on opening all the infected plains in the first operation, in addition to the usage of packed gauze into the cavities. However, this method alone will not provide an effective way to complete recovery. Therefore, we suggest sealed irrigation as an adjuvant therapy to the surgical debridement to maximize necrotizing tissue removal and subsequent healing.

## 4 Conclusion

We have reported a case of necrotizing fasciitis of the abdominal wall and the back in a young patient with no prior medical history, who was treated with surgical debridement and sealed irrigation technique. NF is a rapidly spreading and fatal disease, which requires prompt recognition of signs and symptoms, targeted diagnostic testing, and timely treatment to avoid complications. Sealed irrigation technique is an effective method to manage NF. Furthermore, it reduces the hospitalization duration by continuously removing the necrotic and infected material that hinder tissue healing. Larger studies should be conducted to determine the effectiveness of our technique.

## Provenance and peer review

Not commissioned, externally peer reviewed.

## Ethics approval

No ethical approval necessary.

## Consent

Written informed consent was obtained from the parents of the patient for publication of this case report and accompanying images. A copy of the written consent is available for review by the Editor-in-Chief of this journal on request.

## Availability of data and material

Data mentioned in this case report are available to the reviewers if required.

## Funding

No financial support.

## Author contribution

[Ayham Sammoni], [Okbah Mohamad], [Ali Abdalah], and [Monaf Borhan Alghazali] performed a literature review and wrote the manuscript. [Okbah Mohamad] managed the team. [Maen Al-Aissami] supervised the scientific validity of medical information and diagnosed the case. All authors approved the final manuscript.

## Registration of research studies

1. Name of the registry:

None.

2. Unique Identifying number or registration ID:

None.

3. Hyperlink to your specific registration (must be publicly accessible and will be checked): None.

## Guarantor

Maen Al-Aissami.

## Declaration of competing interest

The authors declare that they have no competing interests.

## Abbreviations

NF: Necrotizing fasciitis
GAS: group A Streptococcus

## References

[1] By L.E.O.N.L. (2020). CHEN DA-BFFFBFPRCACTMA-B. Necrotizing fasciitis: a comprehensive review. Nursing l Volume.

[2] Sarani B., Strong M., Pascual J., Schwab C.W. (2009). Necrotizing fasciitis: current concepts and review of the literature. J. Am. Coll. Surg..

[3] Kapp D.L., Rogers M., Hermans M.H.E. (2018 Dec 1). Necrotizing fasciitis: an overview and 2 illustrative cases. Int. J. Low. Extrem. Wounds.

[4] Rebai L., Daghmouri A., Boussaidi I. (2018 Jan 1). Necrotizing fasciitis of chest and right abdominal wall caused by acute perforated appendicitis: case report. International Journal of Surgery Case Reports.

[5] Rukshini Puvanendran MMed (2009 Oct). FCFP MB BS Jason chan Meng Huey MB BS Shanker Pasupathy FRCS MB BS. Clinical Review Necrotizing fasciitis.

[6] Stevens D.L., Bryant A.E., Longo D.L. (2017 Dec 7). New England Journal of Medicine.

[7] Agha R.A., Franchi T., Sohrabi C., Mathew G., for the SCARE Group (2020). The SCARE 2020 guideline: updating consensus surgical CAse REport [SCARE] guidelines. Int. J. Surg..

[8] Wang J.M., Lim H.K. (2014 Mar). Necrotizing fasciitis: eight-year experience and literature review. Braz. J. Infect. Dis..

[9] Young M.H., Aronoff D.M., Engleberg N.C. (2005). Necrotizing fasciitis: Pathogenesis and treatment. Expert Rev. Anti-infect. Ther..

[10] Morgan M.S. (2010). Diagnosis and management of necrotising fasciitis: a multiparametric approach. J. Hosp. Infect..

[11] Alayed K al, Tan C., Daneman N. (2015 Jul 1). Red flags for necrotizing fasciitis: a case control study. Int. J. Infect. Dis..

[12] Anaya D.A., Dellinger E.P. (2007 Mar 1). Necrotizing soft-tissue infection: diagnosis and management. Clin. Infect. Dis..

[13] Misiakos E.P., Bagias G., Patapis P., Sotiropoulos D., Kanavidis P., Machairas A. (2014).

[14] Zhou E hui, Liu S ru, ming Zhu H., liang Yi H., Chen X ping (2021 Aug 1). Management and postoperative use of double-cannula irrigation–drainage tube in cervical necrotizing fasciitis: a Chinese single-institution experience of 46 patients. Eur. Arch. Oto-Rhino-Laryngol..

